# Epidemiological and transcriptome data identify potential key genes involved in iron overload for type 2 diabetes

**DOI:** 10.1186/s13098-023-01110-0

**Published:** 2023-06-21

**Authors:** Xuekui Liu, Xiu Hong, Shiqiang Jiang, Rui Li, Qian Lv, Jie Wang, Xiuli Wang, Manqing Yang, Houfa Geng, Yang Li

**Affiliations:** 1 Department of Central Laboratory, Xuzhou Central Hospital, Xuzhou, China; 2 Department of Anesthesiology, Xuzhou Renci Hospital, Xuzhou, China; 3 Department of Endocrinology, Xuzhou Central Hospital, Xuzhou, China

**Keywords:** Iron overload, Type 2 diabetes, Insulin secretion, Transferrin receptor, Solute carrier family-11 member-2

## Abstract

**Background:**

Many previous studies have reported the association between iron overload (IO) and type 2 diabetes mellitus (T2DM). However, the underlying molecular mechanism is not clear.

**Methods:**

Epidemiological data from the National Health and Nutrition Examination Survey 2017–2018 (NHANES) was used to systematically explore the association between IO and diabetes. Furthermore, transcriptome data from Gene Expression Omnibus (GEO) were analyzed using bioinformatics methods to explore the underlying functional mechanisms at the molecular level.

**Results:**

Data from NHANES showed a “W” shape relationship between serum iron (frozen) and the risk of diabetes (P < 0.001) as well as a “∧” shape correlation between serum unsaturated iron binding capacity (UIBC) and the risk of diabetes (P = 0.007). Furthermore, the serum iron (frozen) was positively associated with fasting plasma glucose and HOMAB (P < 0.05), and UIBC was positively associated with fasting insulin (P < 0.05). Transcriptome data showed that two IO-related genes [Transferrin receptor (TFRC) and Solute carrier family-11 member-2 (SLC11A2)] were down-regulated in T2DM. The correlation analysis showed that expression levels of TFRC and SLC11A2 were significantly and positively correlated with genes involved in insulin secretion (P < 0.05). Protein–protein interaction network analysis showed that TFRC and SLC11A2 interacted with four key genes, including VAMP2, HIF1A, SLC2A1, and RAB11FIP2.

**Conclusion:**

We found that IO status was associated with increased FPG and aggravated HOMAB, and two IO-related genes (TFRC and SLC11A2) might induce the occurrence of T2DM by influencing insulin secretion, which provides potential therapeutic targets for T2DM patients.

## Introduction

Iron is an essential element of the body and plays a crucial role in cellular processes, such as nucleic acid repair, cellular respiration and DNA synthesis [1]. Iron homeostasis of the body contributes to oxygen transport and supply of erythrocytes [2]. Deficiency or overload of iron can result in abnormal metabolism and accelerate the onset of chronic diseases, such as hereditary hemochromatosis (HH) [3], diabetes [4], liver disease [5], and cardiovascular disease [6]. In recent years, a number of epidemiological studies reported that iron level was associated with diabetes and found that iron overload (IO) was an independent risk factor of type 2 diabetes mellitus (T2DM) [4, 7, 8]. Diabetes is characterized by insulin resistance and insufficient insulin secretion, which is caused by obesity, metabolic syndrome, impaired β-cell function and other risk factors [9]. Liver is an important organ for the storage of iron, and plays a crucial role in iron metabolism. IO status in the liver can lead to many diseases [10], such as non-alcohol fatty liver disease and liver insulin resistance. Previous epidemiological studies reported that IO increases the risk of insulin resistance, indicating the linkage between IO and diabetes [11]. Although previous studies confirmed that IO is a risk factor for diabetes, the associated pathological mechanism is not clear.

National Health and Nutrition Examination Survey (NHANES) is a program of studies designed to assess the health status of adults and children in the United States. This survey has detected the iron status and diabetes status of participants from 2017 to 2018. The large sample size, as well as detailed documentation of clinical indicators, provide strong support for finding disease risk factors. For example, Jun et al. [12] assessed total usual nutrient intakes, Healthy Eating Index-2015 (HEI-2015) scores, and nutritional biomarkers by food security status, sex, and age among US children based on NHANES. They found the association between food insecurity and compromised intake of some micronutrients, especially among adolescent girls. Mei et al. [13] developed a physiological-based method to determine NHANES-based thresholds for iron deficiency in children and non-pregnant women. However, few researchers investigated the association between iron level and diabetes using NHANES. Besides, such studies only find some phenomenon or association by epidemiological analysis and lack the elaboration of molecular mechanisms. The rapid development of sequencing technology and bioinformatic methods provide an efficient means for the in-depth study of molecular mechanisms. For example, Li et al. [14] found that lncRNA XIST promotes iron overload and iron overload-related T2DM initiation and development through inhibition of activin receptor-like kinase 2 expression by sponging miR-130a-3p.

In this study, by comprehensively analyzing the epidemiological data and transcriptome data, we aimed to systematically expound the association between iron overload and T2DM, and further explored the underlying molecular mechanism and key genes involved in T2DM progression, which provides potential therapeutic targets for T2DM patients.

## Subjects and methods

### Subjects of epidemiological investigation

We downloaded the NHANES 2017 ~ 2018 dataset from the web of https://wwwn.cdc.gov/nchs/nhanes/continuousnhanes/default.aspx?BeginYear = 2017. In 2017–2018, 16,211 persons were selected for NHANES from 30 different survey locations. Of those selected, 9,254 completed the interview, and 8,704 were examined. Of the examined participants, 5,922 persons were detected with iron deficiency in their serum iron frozen by mobile examination centers, and 8,709 answered the question “{have you/has SP}/{Have you/Has SP}} ever been told by a doctor or health professional that {you have/{he/she/SP} has} diabetes or sugar diabetes?”. We excluded individuals with age  < 18 years, pregnancy, used marijuana or hashish, and persons with serum iron frozen below the lower detection limit. Finally, a total of 2,411 participants were included in this present study, of which 363 suffered from diabetes and 2,048 were non-diabetics.

### Exposure factor and outcome variables

Iron (frozen) and unsaturated iron binding capacity (UIBC) were measured by Roche cobas 6000 (c501 module) analyzer, and the laboratory quality assurance and monitoring were controlled by NHANES and information published online (https://wwwn.cdc.gov/nchs/data/nhanes/2017-2018/manuals/2017_MEC_Laboratory_Procedures_Manual.pdf). Total iron binding capacity (TIBC) was calculated by iron (frozen) and UIBC; the formula used was TIBC = [serum iron(frozen) + serum UIBC]. Transferrin saturation (%) value was estimated by serum iron (frozen) and serum (UIBC); the equation used was Transferrin saturation (%) = [serum iron(frozen)/serum UIBC] × 100. Using NHANES 2017 ~ 2018 questionnaire data and laboratory data, we assessed diabetes status by the question “Doctor told you have diabetes,” fasting plasma glucose (FPG) concentration, and glycohemoglobin level. As secondary outcomes, FPG, fasting insulin (Fins), and homeostasis model assessment of β-cell function (HOMAB) were detected.

### Covariates variables

Independent relationship between iron load and diabetes were inspected by selecting some covariates, such as the risk of diabetes, and confirmed by previous studies into the final analysis. The covariates included race, sex, age, body mass index (BMI), systolic blood pressure (SBP), diastolic blood pressure (DBP), total cholesterol (TC), triglyceride (TG), lower density lipoprotein (LDL), higher density lipoprotein (HDL), HOMAB, and smoking status (whether the participants smoked at least 100 cigarettes in life (Yes/No)) and alcohol consumption (whether the interviewee even had a frequent drink of any kind of alcohol levels).

### Microarray-based gene expression profiling

Genome-wide liver expression profiles of mice of the B6 and D2 genetic backgrounds (GSE10421) [15] subjected to iron-balanced or -enriched diets were generated using the Agilent Whole Genome microarrays and were attained from the GEO database. All the analyses were performed using Bioconductor, an open source software for the analysis of genomic data rooted in the statistical computing environment R. Raw data were processed by background correction and quantile normalization using the R/Bioconductor. Differential expression analysis was performed using the “limma” package to find differentially expressed genes (DEGs) between iron-balanced and -enriched diets for mice of the B6 and D2, respectively. Another gene expression cohort [16] (GSE164416) containing 39 T2DM and 18 normal samples was downloaded from GEO. The DEGs between T2DM and normal samples were identified using the edgeR package. The threshold was set as p < 0.05.

### Clustering and functional enrichment analyses

The pheatmap clustering algorithm was used to draw heat maps of DEGs. Sample similarity was estimated by the Euclidean distance based on the expression measurements of the consistent DEGs in B6 and D2 mice. In order to explore the biological functions in which the DEGs might be involved, we selected the consistent DEGs in B6 and D2 mice to make the functional enrichment analysis using the R package "clusterProfiler" for Gene Ontology (GO, involving three categories: biological processes, molecular function, and cellular components) and Kyoto Encyclopedia of Genes and Genomes (KEGG). The criterion for significant enrichment was p < 0.05.

### Construction of the protein–protein interaction (PPI) network

The Search Tool for the Retrieval of Interacting Genes/Proteins (STRING) database (https://string-db.org/) was used to construct the PPI network. This database has a comprehensive score for each PPI relationship pair that is distributed between 0 and 1; the higher the score, the more reliable the PPI relationship. In this study, PPI relationship pairs were selected by applying a medium confidence criterion (confidence score ≥ 0.4). The Cytoscape software was used to visualize the PPI network. Using cytoHubba application,the top ten hub genes with high degrees of connectivity were identified.

### Statistical methods

We utilized R4.1.3 software to manage our data. R packages including “limma”, “tidyverse” and “rms” were performed to explore the association between iron indices and the risk of diabetes. Clinical characteristics and iron indices of the diabetes and non-diabetes groups were compared using a two-sample t-test for variables with normal distribution, a χ^2^ test for variables with count data, and a non-parametric test for variables with non-normal distribution. General linear mixed model was performed to analyze the association between iron indices and the risk of diabetes with glucose indices, such as FPG, Fins and HOMAB. Restricted cubic spline plots were used to show the non-linear relationship between iron indices and the risk of diabetes and glucose indices.

## Results

### The clinical characteristics of individuals in 2017–2018 NHANES

**Fig. 1:**
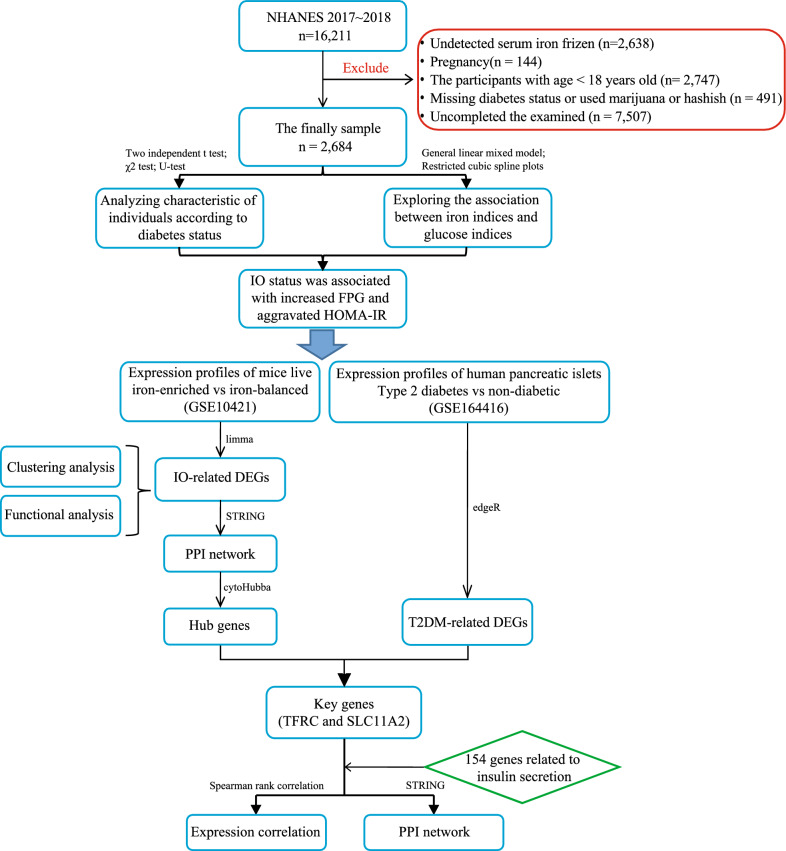
The flowchart for this study

**Fig. 2:**
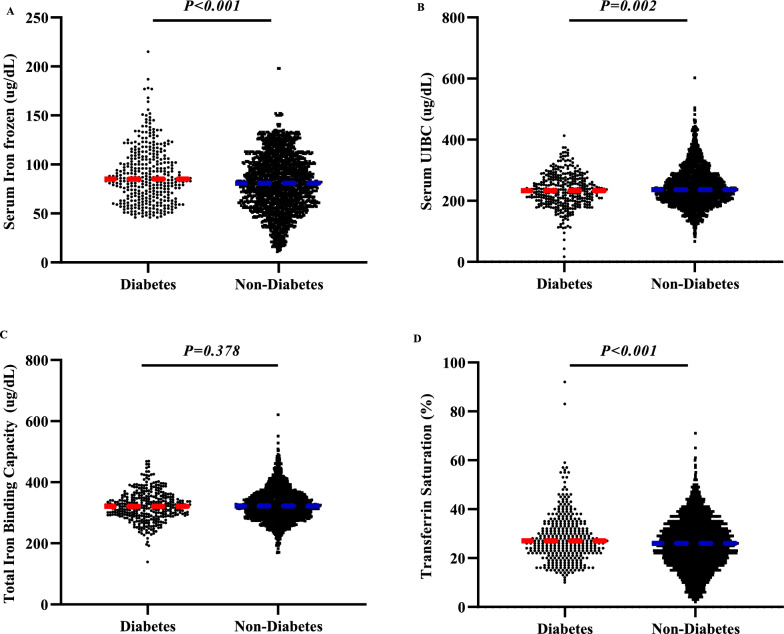
The serum iron status component in different diabetes status. **A** Serum iron frozen in different diabetes status; **B** Serum Unsaturated Iron Binding Capacity (UIBC) in different diabetes status; **C** The total Iron Binding Capacity in different diabetes status; **D** The transferrin saturation in different diabetes status

The flowchart for this study is shown in Fig. 1. According to diabetes status, we compared the differences in clinical characteristics between diabetes and non-diabetes groups. The results showed that sex, age, BMI, SBP, TC, TG, HDL, LDL, FPG, Fins, HOMAB and smoking status have significant differences in the two groups, while DBP, race and alcohol consumption did not show a significant difference between two groups (Table 1). As shown in Fig. 2, the levels of serum iron (frozen), serum UIBC and transferrin saturation showed significant difference between two groups. Participants with diabetes had a higher serum iron (frozen) level (Fig. 2A) and transferrin saturation level (Fig. 2D) and a lower serum UIBC (Fig. 2B). Total iron binding capacity (Fig. 2C) did not differ significantly between two groups.

**Table 1:** The clinical characteristic of individuals in 2017–2018 NHANES

Variables	Non-diabetes	Diabetes	P
n	2048	363	
Gender			
Male	917 (81.9%)	203 (18.1%)	< 0.001
Female	1131 (87.6%)	160 (12.4%)	
Age(years)	42.55 ± 20.71	63.54 ± 12.41	< 0.001
BMI(kg/m^2^)	28.61 ± 7.42	32.24 ± 7.74	< 0.001
SBP(mmHg)	121.77 ± 19.31	133.60 ± 20.69	< 0.001
DBP(mmHg)	70.55 ± 13.06	69.91 ± 14.36	0.418
TC(mmol/L)	4.71 ± 1.04	4.45 ± 1.16	< 0.001
TG(mmol/L)	1.07 ± 0.67	1.47 ± 0.72	< 0.001
HDL(mmol/L)	1.40 ± 0.38	1.27 ± 0.35	< 0.001
LDL(mmol/L)	2.81 ± 0.91	2.51 ± 1.01	< 0.001
FPG(mmol/L)	5.74 ± 1.05	8.82 ± 3.23	< 0.001
Fins(uU/mL)*	2.31 ± 0.71	2.66 ± 0.89	< 0.001
HOMAB	93.76 (61.79 ~ 148.58)	61.80 (33.81 ~ 108.32)	< 0.001
Race			
Mexican American	285 (80.1%)	71 (19.9%)	0.098
Other hispanic	185 (83.3%)	37 (16.7%)	
Non-hispanic white	698 (85.7%)	116 (14.3%)	
Non-hispanic black	490 (86.7%)	75 (13.3%)	
Non-hispanic Asian	263 (85.7%)	44 (14.3%)	
Other race	127 (86.4%)	20 (13.6%)	
Smoking status			
Yes	662 (77.5%)	192 (22.5%)	< 0.001
No	1085 (86.5%)	170 (13.5%)	
Alcohol consumption			
Yes	1456 (82.4%)	311 (17.6%)	0.117
No	177 (86.8%)	27 (13.2%)	

^*^The variable was transferred by ln

*BMI* body mass index, *SBP* systolic blood pressure, *DBP* diastolic blood pressure, *TC* total cholesterol, *TG* triglyceride, *LDL* lower density lipoprotein, *HDL* higher density lipoprotein, *FPG* fasting plasma glucose, *Fins* fasting insulin, *HOMAB* Homeostasis model assessment β cell function; Smoking status: whether the participants smoked at least 100 cigarettes in life (Yes/No). Alcohol consumption: whether the interviewee ever had a drink of any kind of alcohol. We compared clinical characteristics and iron indices between the diabetes and non-diabetes groups using a two-sample t-test for variables with normal distribution, χ2 test for variables with count data, and a non-parametric test for variables with non-normal distribution

### The iron indices associated with the risk of diabetes

**Fig. 3:**
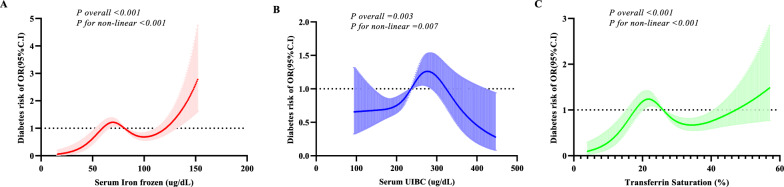
The relationship between serum iron status components and the risk of diabetes. **A** The relationship between serum iron frozen and the risk of diabetes; **B** The relationship between serum UIBC and the risk of diabetes; **C** The relationship between transferrin saturation and the risk of diabetes

A non-linear relationship was noted between iron indices and the risk of diabetes in US adults. As shown in Fig. 3, a “W” shape relationship was observed between serum iron (frozen) and the risk of diabetes (P for non-linear < 0.001), and a “∧” shape correlation between serum UIBC and the risk of diabetes (P for non-linear = 0.007). Transferrin saturation, estimated by serum iron (frozen) and serum UIBC, was also associated with the risk of diabetes; the P-value for non-linear  < 0.001.

### The relationships between iron indices and glucose indices in USA adults

**Fig. 4:**
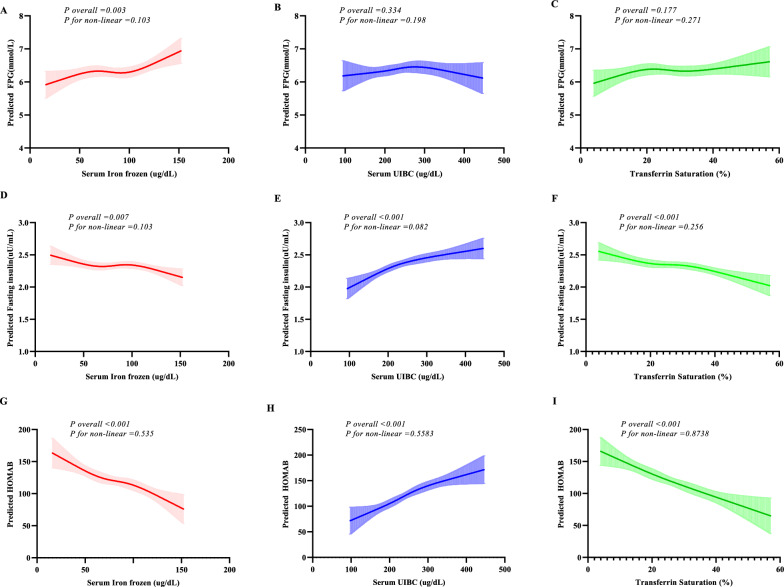
The association between serum iron status components and glucose metabolic variables. **A**–**C** The association between serum iron status components and the predicted value of fasting plasma glucose; **D**–**F** The association between serum iron status components and the predicted value of fasting insulin; **G**–**I** The association between serum iron status components and the predicted value of HOMAB

Performing general linear mixed model using ordinary least squares, we explored the correlation between iron indices and glucose indices. The results show that serum iron (frozen) was positively associated with FPG (Fig. 4A) and HOMAB (Fig. 4G) and negatively related to Fins (Fig. 4D). Serum UIBC was positively associated with Fins (Fig. 4E) and HOMAB (Fig. 4H). Transferrin saturation was associated with Fins (Fig. 4F) and HOMAB (Fig. 4I), but not related to FPG (Fig. 4C).

### Identification of differentially expressed genes related to iron overload

**Fig. 5:**
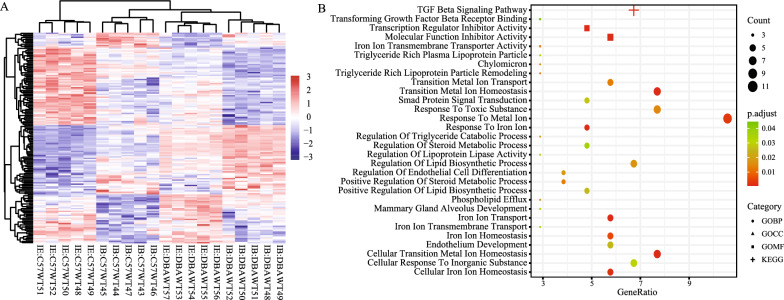
Heat maps **A** and functional enrichment **B** based on differentially expressed genes

Based on the expression profiles obtained from the GEO database, we initially performed differential expression analysis by comparing gene expression between iron-balanced and -enriched diets for mice of the B6 and D2 respectively. Using a cutoff criteria of P < 0.05, 2808 and 1205 DEGs were identified in two kinds of mice of the B6 and D2 respectively. A total of 155 DEGs (including 62 upregulated genes and 93 downregulated genes) were concordance. The concordance score was 97.8% (binomial test, P < 0.001). Based on expression profiles of the concordance DEGs, heat map was shown using the pheatmap clustering algorithm (Fig. 5A).

### Functional enrichment and protein–protein interaction network of differentially expressed genes

The concordance DEGs were analyzed to explore the underlying functions and pathways. The significant terms emerging from the GO enrichment analysis were shown in Fig. 5B. GO annotation showed that DEGs were significantly enriched in many iron-related terms, such as cellular iron ion homeostasis, cellular transition metal ion homeostasis, iron ion transmembrane transport and response to iron ion. KEGG analysis showed that DEGs were significantly involved in TGF beta signaling pathway.

**Fig. 6:**
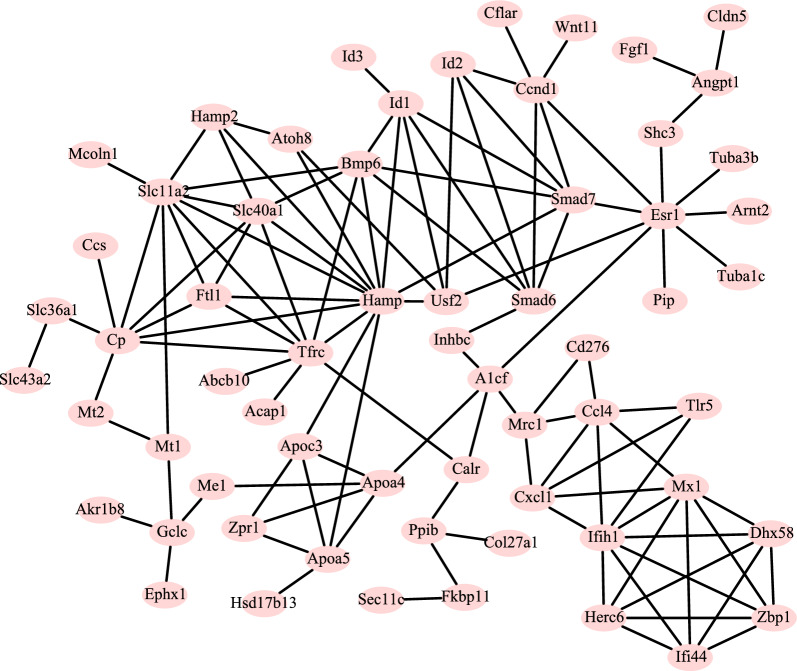
The Protein–Protein interaction (PPI) network based on differentially expressed genes

Using the STRING database, we constructed the PPI network based on the concordance DEGs. With a cutoff criterion of interaction score  > 0.4, a PPI network containing 124 interactions was constructed after removing unconnected nodes. As shown in Fig. 6, the PPI network was visualized by the Cytoscape software. Furthermore, the cyto-Hubba application was used to select the potential key genes in the network. The top ten hub genes with high degrees of connectivity were identified, including Hamp, Tfrc, Slc11a2, Ifih1, Cp, Slc40a1, Mx1, Ftl1, Dhx58 and Herc6 (Table 2).

**Table 2:** The expression difference of ten hub genes in male mice of the C57BL/6 and DBA/2

GeneNames	C57BL/6			DBA/2		
	logFC	t	P value	logFC	t	P value
Hamp	1.77	7.74	1.35E-05	2.11	3.79	3.28E-03
Tfrc	−1.60	−8.11	8.88E-06	−1.24	−7.71	1.26E-05
Slc11a2	−0.47	−5.59	2.11E-04	−0.26	−3.55	4.94E-03
Ifih1	−0.39	−4.53	1.03E-03	−0.28	−2.50	0.030
Cp	0.36	2.91	0.015	0.48	3.46	5.73E-03
Slc40a1	0.66	4.71	7.67E-04	0.39	2.96	0.014
Mx1	−0.68	−2.67	0.023	−0.38	−2.25	0.047
Ftl1	0.59	5.94	1.29E-04	0.48	3.55	4.96E-03
Dhx58	−0.79	−4.09	2.08E-03	−0.41	−2.48	0.031
Herc6	−0.44	−3.25	8.36E-03	−0.47	−2.54	0.028

### Association between iron overload-related genes and insulin secretion in T2DM

**Fig. 7:**
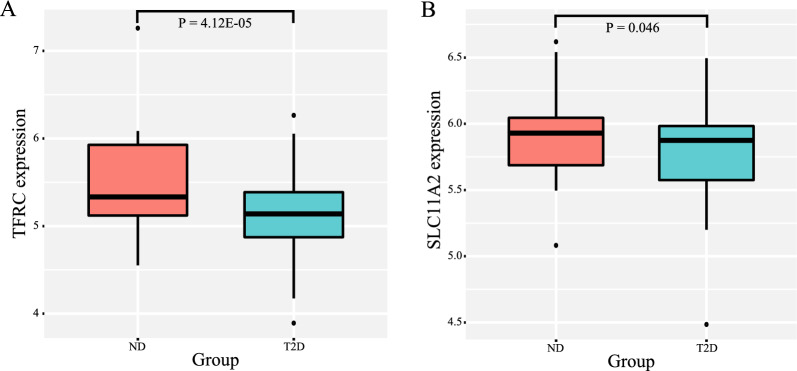
Expression levels between type 2 diabetes (T2D) and non-diabetic (ND) for TFRC and SLC11A2, respectively

To explore the association with T2DM, we firstly tested whether the hub genes were differentially expressed. After removing unmatched genes, the results showed that two genes [including transferrin receptor (TFRC) and solute carrier family-11 member-2 (SLC11A2)] were both significantly down-regulated in T2DM samples when compared with normal samples, indicating that the downregulation of two key genes might cause IO and further induce the occurrence of T2DM (Fig. 7).

**Fig. 8:**
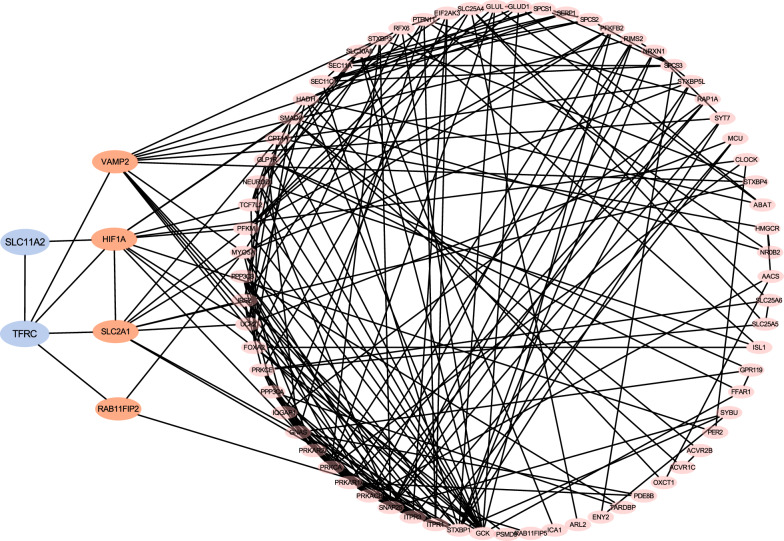
Interaction between two IO-related genes (TFRC and SLC11A2) and genes related to insulin secretion

Epidemiological analysis showed a negative relationship between serum iron (frozen) and Fins, indicating that IO might affect insulin secretion and eventually induce diabetes. To explore the association between two IO-related genes (TFRC and SLC11A2) and insulin secretion, we selected 154 genes related to insulin secretion from GO annotation and calculated the expression correlations with TFRC and SLC11A2 using Spearman rank correlation, respectively. The results showed that expression levels of 92 out of 154 genes were significantly and positively correlated with TFRC gene expression (binomial test p = 0.019). Similarly, expression levels of 113 out of 154 genes were significantly and positively correlated with SLC11A2 gene expression (binomial test p < 0.001). Such results showed a close association between key genes (TFRC and SLC11A2) and insulin secretion. To further explore the association between IO and insulin secretion, we constructed the PPI network using the STRING database. After removing the discrete points, TFRC and SLC11A2 were observed to interact with many key genes (such as VAMP2, HIF1A, SLC2A1 and RAB11FIP2) related to insulin secretion (Fig. 8). These results indicated that IO-related genes (TFRC and SLC11A2) might induce diabetes by affecting many key genes related to insulin secretion.

## Discussion

Using NHANES 2017 ~ 2018 dataset, GSE10421 and GSE164416, we found that IO increased the FPG levels and reduced HOMAB levels in adults from the USA, and a non-linear relationship was observed between iron levels and the risk of diabetes. We also found that two IO-related genes including TRFC and SLC11A2, were down-regulated in patients with diabetes and were closely associated with genes related to insulin-secretion. Those findings confirmed that IO status was contributed to the development of diabetes via affecting insulin secretion.

In a total of 2,411 participating adults from the USA, we found that the participants with higher iron level have a higher prevalence rate of diabetes, although the relationship was not linear. The results agreed with those reported in previous studies. For example, in the Chinese Health and Nutrition Survey Cohort study, Gao H et al. [17] found IO is associated with diabetes, and this relationship may be associated with insulin resistance indirectly induced by IO. Jiang L et al. [18] also found that circulating ferritin levels were independently related with increase diabetes. In our current study, we found that serum iron frozen was positively associated with the risk of diabetes and FPG and negatively related with Fins and HOMAB. We speculated that IO status could inhibit the secretion of insulin and elevate the serum glucose.

Based on the findings from the epidemiological analysis, we further explored the underlying IO-related molecular mechanisms associated with T2DM using transcriptomic data. We found that two IO-related genes (TFRC and SCL11A2) were also significantly down-regulated in patients with T2DM, indicating that the downregulation of the two IO-related genes might play key roles in T2DM. TFRC constitutes the major receptor by which most cells take up iron [19, 20]. Professor José Manuel Fernández-Real [21] found that the frequency of the G allele at the position 210 of the TFRC gene was significantly higher in patients with T2DM and sixteen other TFRC SNPs were also associated to T2DM according to the Welcome Trust Case Control Consortium data, demonstrating that TFRC gene polymorphisms are associated with T2DM. SLC11A2 is a key player in transporting ferrous iron and some divalent metal ions throughout the plasma membrane and across endosomal membranes [22, 23]. Cansu Ozbayer et al. [24] found that the homozygous CC genotype for SLC11A2 gene variants IVS4 + 44C/A showed a significant correlation with the risk of T2DM. These studies confirmed that dysregulation of TFRC and SLC11A2 is associated with iron overload and increased the risk of T2DM.

Epidemiological analysis showed a negative correlation of serum iron (frozen) with Fins and HOMAB, indicating that IO might affect insulin secretion and eventually induce diabetes. In our study, we calculated the expression associations between two IO-related genes (TFRC and SLC11A2) and 154 genes related to insulin secretion using Spearman rank correlation and further constructed a PPI network. The results showed that most genes related to insulin secretion were significantly and positively correlated with TFRC or SLC11A2 expression. Moreover, four key genes related to insulin secretion interact directly with either TFRC or SLC11A2 in PPI network, indicating the close relationship between iron overload and insulin secretion. Vesicle-associated membrane protein 2 (VAMP2), as a soluble N-ethylmaleimide-sensitive factor attachment protein receptor, plays crucial roles in insulin secretion [25–27]. Barillaro M et al. [28] found that COL IV cells display prominent colocalization of Snap25 and Vamp2 distributed at the cell membrane and in the cytoplasm, correlating with an increase in insulin secretion rate compared to the control during glucose stimulated insulin secretion, providing evidence of β1-integrin influencing the colocalization of Snap25 and Vamp2 in connection to increased insulin secretion rates. Hypoxia-inducible factor 1A (HIF1A) is involved in β-cell dysfunction and is a mediator of T2DM [29, 30]. Wang et al. [31] demonstrated that glucotoxicity-induced insulin secretion defects in INS-1E cells could be mediated by HIF1A via the down-regulation of Calcium/calmodulin-dependent serine protein kinase (CASK). Solute carrier family 2 member 1 (SLC2A1 or GLUT1) plays a crucial role in glucose transport in human insulin-secreting β-cells [32–34]. Yang et al. [35] found that the small GTPase Rheb1 facilitates glucose-stimulated insulin secretion in human or mouse islets by upregulating the expression of GLUT1 or GLUT2, respectively. RAB11 family interacting protein 2 (RAB11FIP2), as a conserved protein and effector molecule for the small GTPase Rab11, is found to play important roles in tumor progression and metastasis [36–38]. However, few researchers reported the association between RAB11FIP2 and insulin secretion. In current study, we found that TFRC and SLC11A2 could interact directly with these four genes, indicating that the dysregulation of TFRC and SLC11A2 might induce the occurrence of T2DM by regulating insulin secretion. The regulatory mechanism requires further experimental investigation in future studies.

There are some limitations in this study. Firstly, we used NHANES 2017–2018 dataset as epidemiology analysis data in current study. However, all participants were from the USA, which may be limited extrapolation of statistical findings. Secondly, the bioinformatic datasets, which are from GEO, need to be verified using our independent experiment. Besides, using DEGs from two different species (mice and humans) for comparison is not entirely acceptable. Further study is needed to collect serum iron information and pancreatic islets of human donors and then identify the DEGs related to IO and T2DM.

## Conclusion

In summary, we found that IO status was associated with increased FPG and aggravated HOMAB, and two IO-related genes (TFRC and SLC11A2) might induce the occurrence of T2DM by influencing insulin secretion, which provides potential therapeutic targets for T2DM patients.

## Data Availability

All data generated or analyzed during this study are included in this manuscript.

## Abbreviations

IO: Iron overload
T2DM: Type 2 diabetes mellitus
NHANES: National Health and Nutrition Examination Survey
GEO: Gene expression omnibus
UIBC: Unsaturated iron binding capacity
TRFC: Transferrin receptor
SLC11A2: Solute carrier family-11 member-2
PPI: Protein–protein interaction
HOMAB: Homeostasis model assessment of β-cell function
TIBC: Total iron binding capacity
BMI: Body mass index
SBP: Systolic blood pressure
DBP: Diastolic blood pressure
TC: Total cholesterol
TG: Triglyceride
LDL: Lower density lipoprotein
HDL: Higher density lipoprotein (HDL)
DEGs: Differentially expressed genes
